# Rho/ROCK Pathway as a Therapeutic Target in Multiple Diseases

**DOI:** 10.53941/ijddp.2025.100018

**Published:** 2025-09-05

**Authors:** Zixiu Cheng, Shannon Erhardt, Jun Wang

**Affiliations:** 1 Department of Pediatrics, McGovern Medical School at UTHealth, The University of Texas Health Science Center at Houston, Houston, TX 77030, USA; 2 Graduate School of Biomedical Sciences, The University of Texas Health Science Center at Houston, Houston, TX 77030, USA

**Keywords:** Rho, ROCK, drug-targeting, fibrosis

## Abstract

The Rho/ROCK (Rho-associated coiled-coil-containing protein kinase) signaling pathway plays a pivotal role in regulating diverse cellular processes, including cytoskeletal organization, cell migration, proliferation, and apoptosis. Dysregulation of this pathway has been implicated in the pathogenesis of various diseases, such as cardiovascular disorders, cancer, neurological conditions, and fibrotic diseases. Accumulating evidence supports the therapeutic potential of targeting Rho/ROCK, with several inhibitors currently under investigation or in clinical use. This review summarizes the molecular mechanisms underlying Rho/ROCK signaling, explores its involvement in disease progression, and discusses recent advances in the development of and the clinical application of ROCK inhibitors as promising therapeutic agents.

## 1. Rho/ROCK Pathway

### 1.1. Function and Regulation

The Rho family of small GTPases, particularly RhoA, and their major downstream effector kinases, Rho-associated coiled-coil containing protein kinase 1 and 2 (ROCK1 and ROCK2), form a key signaling component that modulates actin cytoskeletal dynamics and influences a broad range of physiological and pathological processes [1–7]. Since its identification, Rho/ROCK signaling has been recognized as a central regulator in multiple systems, including the cardiovascular, nervous, immune, and renal systems [4,8–15]. Aberrant activation of the Rho/ROCK pathway has been linked to various human diseases. In cardiovascular pathology, for instance, ROCK contributes to endothelial dysfunction, vascular inflammation, and cardiac remodeling [4, 8]. In cancer, the Rho/ROCK pathway plays important roles in tumor development and progression by regulating tumorigenicity, tumor growth, metastasis, angiogenesis, tumor cell death, and chemoresistance [16]. Additionally, ROCK activity is associated with neuronal injury and fibrotic responses in multiple organs [9, 10, 15, 17 – 19]. Given its broad pathological relevance, the Rho/ROCK pathway has emerged as a promising target for pharmacological intervention [20]. While several previous reviews have discussed Rho-targeting therapies [8,11,13,21,22], this review focuses specifically on ROCK inhibitors that are either approved or currently undergoing clinical investigation, highlighting their translational potential in therapeutic applications.

**Figure 1.**
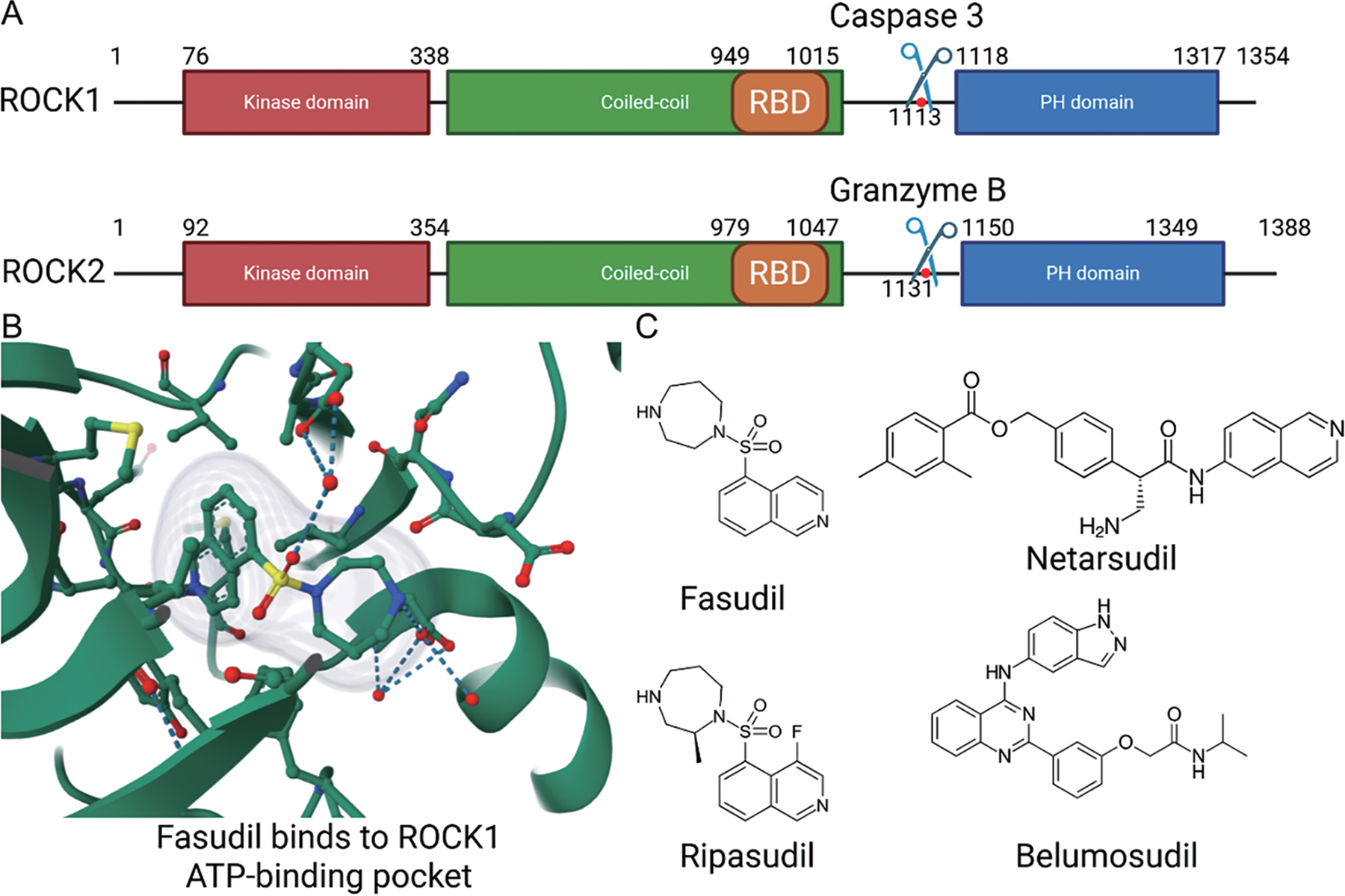
ROCK structure and drug targeting domain. (**A**) Structure of ROCK1 and ROCK2, Scissors located near the C-terminal inhibitory domain of ROCK1 and ROCK2 indicate the sites of proteolytic cleavage by Caspase-3 and Granzyme B, respectively. (**B**) The binding of fasudil to the ROCK1 ATP-binding pocket located in the groove formed by the N-terminal and C-terminal of the kinase domain. (**C**) Structural formulas of approved ROCK inhibitors. All currently available ROCK-targeting drugs act on the kinase domain as ATP-competitive inhibitors.

The Rho-associated kinase ROCK1 and ROCK2 belong to the AGC kinase family, named after its family member protein kinases A, G, and C. As shown in Figure 1A, the ROCK kinases consist of an N-terminal kinase domain, a coiled-coil domain in which the Rho binding domain (RBD) is located, and a C-terminal inhibitory domain, with ROCK1 and ROCK2 sharing ~65% compositional similarity [23]. In their inactive state, ROCKs adopt an auto-inhibited conformation in which the C-terminal region (containing a pleckstrin homology domain) folds back to suppress the kinase activity of the N-terminal catalytic domain [4].

**Figure 2.**
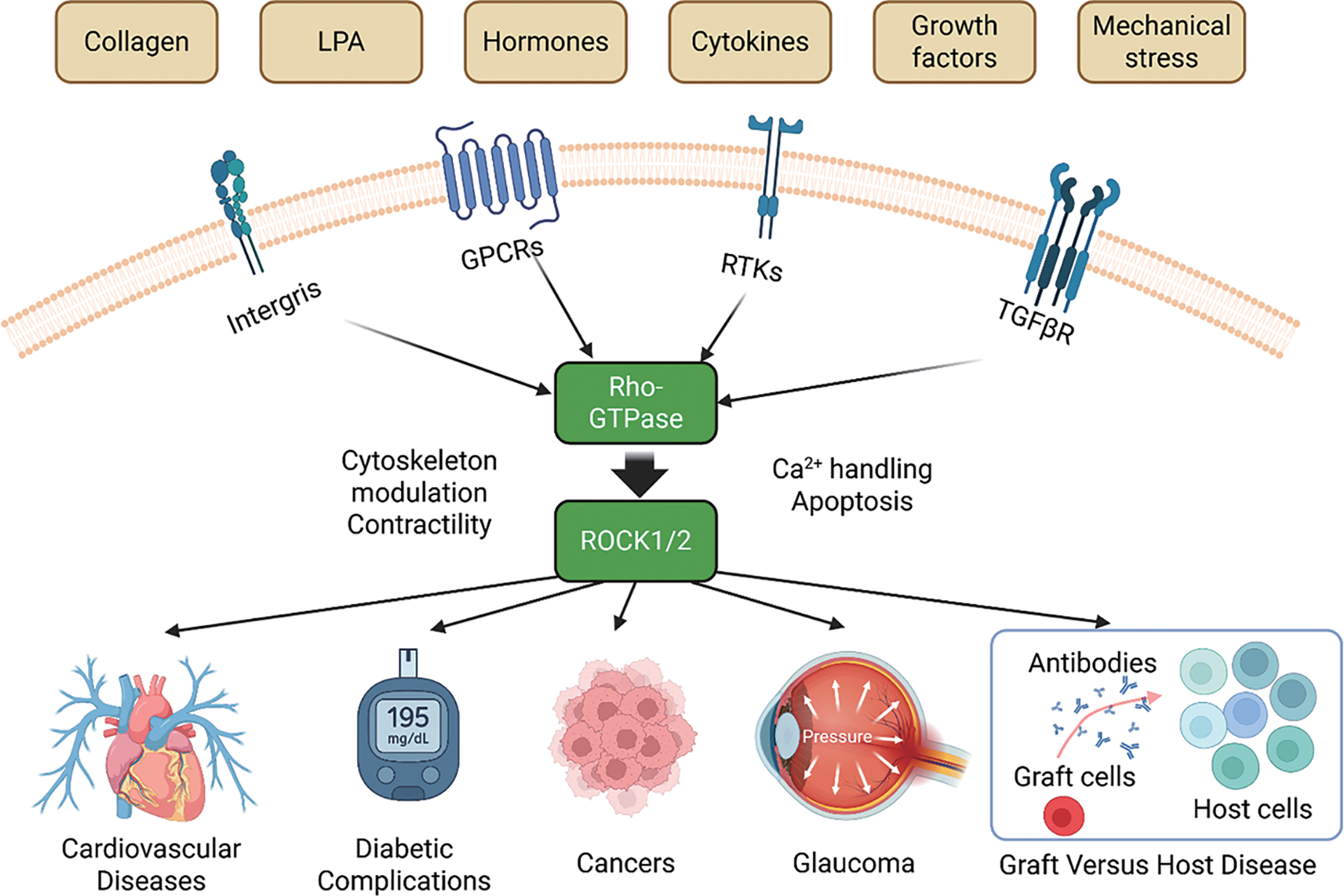
Rho/ROCK pathway and diseases.

As shown in Figure 2, activation of RhoA is triggered by a variety of extracellular signals, including growth factors, cytokines, thrombin, and mechanical stress, which signal through membrane receptors such as G protein-coupled receptors (GPCRs), receptor tyrosine kinases (RTKs), and integrins. These receptors stimulate specific guanine exchange factors (GEFs), such as LARG and p115-RhoGEF, which catalyze the exchange of GDP for GTP on RhoA [24 – 26]. Upon activation, the GTP-bound RhoA directly binds to ROCK’s RBD, inducing a conformational change that releases the auto-inhibitory interaction and activates the kinase domain. This enables ROCK to phosphorylate various downstream substrates, including myosin light chain (MLC), LIM domain kinase (LIMK), and myosin phosphatase targeting subunit (MYPT1), thereby promoting actomyosin contractility and stress fiber formation [5,7,20]. In addition to RhoA-mediated activation, ROCK1 and ROCK2 can also be activated independently through proteolytic cleavage—ROCK1 by caspase-3 during apoptosis or under disease conditions [27], and ROCK2 by granzyme B, released from cytotoxic lymphocytes [28]—which removes their C-terminal inhibitory domain and results in constitutive kinase activity (Figure 1A). Moreover, mechanical cues from the extracellular matrix, such as increased matrix stiffness or integrin engagement, can also promote RhoA activation, thereby linking biophysical stimuli to intracellular contractile signaling via Rho/ROCK [5,24].

### 1.2. ROCK Substrates

Similar to many other AGC family kinases, ROCKs recognize the consensus R/K-X-X-S/T or R/K-X-S/T motif, but some non-consensus sites also exist [29]. The most well-studied ROCK substrates include cytoskeleton-associated proteins such as LIMK and contraction-related proteins like MYPT (MBS) and MLC. During cell morphogenesis and motility, the cytoskeleton is extensively remodeled. ROCK kinases phosphorylate and activate LIMK, which subsequently phosphorylates cofilin, thereby inhibiting its F-actin–depolymerizing activity, thus stabilizing the actin cytoskeleton [30]. MYPT, the regulatory subunit of myosin light chain phosphatase (MLCP), targets PP1c to myosin and facilitates MLCP activity. By promoting MLC dephosphorylation, MLCP counteracts contraction and leads to relaxation of smooth muscle cells [31]. ROCK kinases phosphorylate MYPT1, thereby inhibiting MLCP activity. This inhibition prevents MLC dephosphorylation, leading to enhanced MLC phosphorylation and consequent stress fiber formation, focal adhesion assembly, and cell contraction [5].

Other substrates of ROCK kinases include CPI-17, DAPK3, GFAP, TPPP1, PFN1, FHOD1, JIP-3, PTEN, ERM, adducin, NHE1, and Vimentin. Phosphorylation of CPI-17 enhances its ability to inhibit myosin phosphatase, thereby increasing MLC phosphorylation and promoting smooth muscle contraction. DAPK3 phosphorylation contributes to actomyosin contractility and cell death signaling. Phosphorylation of GFAP and vimentin modifies the assembly and disassembly of intermediate filaments, which is essential for regulating cell shape and migration. ROCK-mediated phosphorylation of PTEN negatively regulates its phosphatase activity, thereby promoting PI3K/AKT signaling and influencing cell survival and polarity. ERM proteins phosphorylation by ROCK induces conformational activation, allowing them to cross-link actin filaments to membrane proteins and stabilize cell surface structure. FHOD1 phosphorylation facilitates its actin nucleation activity and the formation of stress fibers. Phosphorylation of TPPP1 by ROCK prevents its Hdac6 inhibitory activity to enable cells to enter S-phase. JIP-3 phosphorylation enhances its interaction with JNK signaling components, promoting stress response pathways. Phosphorylation of adducin by Rho-kinase plays a crucial role in the regulation of membrane ruffling and cell motility. NHE1 phosphorylation regulates ion exchange activity and regulates intracellular pH during cell migration. These precise substrate-specific modifications underscore the critical role of ROCK in coordinating cytoskeletal architecture, contractility, and intracellular signaling [32–45].

## 2. Rho/ROCK in Disease

### 2.1. ROCK in Cardiovascular Disease

In patients with pulmonary arterial hypertension (PAH), the expression of ROCK2 was found to be higher in pulmonary arterial media and primary pulmonary arterial smooth muscle cells compared to controls. Furthermore, heterozygous ROCK2 deficient mice showed reduced hypoxia-induced pulmonary hypertension (PH) while the ROCK2 overexpression transgenic model had more severe PAH compared to their littermates [46]. Cardiomyocyte specific deletion of ROCK1 or ROCK2 by αMHC-Cre leads to different consequences in the transverse aortic constriction (TAC) induced chronic pressure overload model. Cardiomyocyte-specific ROCK1 deficiency (cROCK1^−/−^) promoted pressure-overload-induced cardiac dysfunction and postcapillary PH, whereas cardiomyocyte-specific ROCK2 deficiency (cROCK2^−/−^) showed opposite results. Pressure overload-induced hypotrophy and fibrosis are enhanced in cROCK1^−/−^ mice while hypotrophy is attenuated in cROCK2^−/−^ mice after TAC [47]. These results indicate that the role for ROCK1 and ROCK2 may be context dependent.

The deletion of both ROCK1 and ROCK2 in the heart decreases fibrosis in an aging mouse model, while the deletion of only ROCK2 leads to increased fibrosis, possibly due to a compensatory upregulation of ROCK1 [48]. Fibroblast-specific deletion of Rock2 attenuates cardiac hypertrophy, fibrosis, and diastolic dysfunction; ROCK2^Postn−/−^ mice had reduced left ventricular (LV) wall thickness and fibrosis, along with improved isovolumetric relaxation in response to angiotensin II (Ang II) compared to the control. Decreased connective tissue growth factor (CTGF) and fibroblast growth factor–2 (FGF2) expression in the hearts of ROCK2^Postn−/−^ mice is also observed. Furthermore, cardiomyocytes incubated with conditioned media from ROCK2-knockdown cardiac fibroblasts exhibited less hypertrophic response, which indicates a non-autonomous effect of ROCK2 in fibroblasts to cardiomyocytes [49].

Atherosclerosis is a central pathological process underlying most cardiovascular diseases. Inhibition of Rho kinase can alleviate atherosclerotic plaque formation or reverse arteriosclerotic coronary lesions in several mouse models [50–52]. In a lysophosphatidic acid-induced model, lack of ROCK2, but not ROCK1, decreases the migration and adhesion of monocytes to endothelial cells, a critical initiating event in the early development of atherosclerosis [53]. ROCK1 cleavage by caspase 3 plays an important role in cardiomyocyte apoptosis in heart failure patients and mouse models [54]. Collectively, these findings highlight the distinct and context-dependent roles of ROCK1 and ROCK2 in pulmonary hypertension, cardiac remodeling, fibrosis, and atherosclerosis, underscoring their potential as differential therapeutic targets in cardiovascular diseases.

### 2.2. ROCK in Diabetes Complications

Many studies have shown that the Rho/ROCK pathway plays a critical role in the pathogenesis of diabetic complications by mediating inflammation, oxidative stress, and endothelial dysfunction [55–58]. ROCK signaling promotes the expression of pro-inflammatory cytokines, enhances reactive oxygen species (ROS) production, and impairs nitric oxide (NO) bioavailability [51,59]. However, the inhibition of ROCK kinase shows a protective effect in diabetic nephropathy, retinopathy, and cardiomyopathy [55,57,58]. Diabetic patients have an increased risk of heart failure and sudden death, and this is partially attributed to diabetic cardiomyopathy [59]. Even though the mechanism underlying the onset of diabetic cardiomyopathy is not fully understood, the overactivation of Rho/ROCK is among the contributors. Knocking out RhoA or ROCK2 protects the diabetic heart from arrhythmogenesis, contractile dysfunction, fibrosis, hypertrophy, and apoptosis [4, 8]. ROCK inhibition also improves contractile function and Ca^2+^ handling in diabetic cardiomyocytes, possibly by downregulating the overactivated CamKII kinase and the downstream phosphorylation of RYR2 [58].

Diabetes also commonly affects the kidneys, leading to a condition known as diabetic kidney disease (DKD). DKD is characterized by persistent albuminuria, a progressive decline in glomerular filtration rate (GFR), and an increased risk of cardiovascular events. It is a leading cause of chronic kidney disease (CKD) and end-stage renal disease (ESRD) worldwide, and has been previously reviewed [55,57]. Recently, novel discoveries regarding the function of ROCK in diabetic nephropathy have been made. In diabetic patients and many diabetic mouse models, ROCK2 levels are upregulated in renal and glomerular cells [55,56]. Ablation of ROCK2 in podocytes prevents diabetic renal damage in mice by promoting PPARα expression and thus improving fatty acid metabolism [60]. Furthermore, ROCK1 has been reported to mediate the mitochondrial fission triggered by hyperglycemia in podocytes and endothelial cells, contributing to diabetic nephropathy [61]. Diabetic patients are prone to developing ulcers, especially in the lower extremities, due to poor circulation and nerve damage [62,63]. In diabetic patients and mouse models, ROCK1 expression is upregulated in wound tissues and ulcers. Inhibition of ROCK enhances wound healing, at least in part, by activating the RIPK4/AMPK signaling pathway [64]. Overall, aberrant activation of the Rho/ROCK pathway contributes to multiple diabetic complications—including cardiomyopathy, nephropathy, retinopathy, and impaired wound healing—through mechanisms involving inflammation, oxidative stress, endothelial dysfunction, metabolic dysregulation, and mitochondrial injury, while ROCK inhibition or selective isoform ablation confers protective effects.

### 2.3. ROCK in Neurological Disorders

ROCK inhibitors have emerged as promising therapeutic agents in the context of neurological injuries and neurodegenerative diseases because they regulate cytoskeletal dynamics, attenuate inflammation, and enhance neuronal survival [9,10,17]. Following central nervous system (CNS) injury, such as spinal cord injury or traumatic brain injury, activation of the Rho/ROCK pathway contributes to axonal growth inhibition, glial scar formation, and neuroinflammation. Pharmacological inhibition of ROCK has been shown to promote axonal regeneration, suppress astrocyte and microglial activation, and improve functional recovery in preclinical models [10]. In neurodegenerative diseases such as Parkinson’s disease (PD), Alzheimer’s disease, and amyotrophic lateral sclerosis (ALS), aberrant ROCK signaling is associated with mitochondrial dysfunction, neuronal apoptosis, and synaptic degeneration [65–67]. ROCK inhibitors, including fasudil and ripasudil, have demonstrated neuroprotective effects in animal models by enhancing neuronal survival, reducing oxidative stress, and preserving synaptic integrity [68,69]. These findings support the therapeutic potential of ROCK inhibition as a multifaceted strategy to counteract neuronal damage and promote regeneration in various neurological conditions.

### 2.4. ROCK in Cancers

The Rho/ROCK signaling pathway is critically involved in multiple aspects of cancer progression, including cell proliferation, migration, invasion, and metastasis [16,70]. ROCK activation enhances actomyosin contractility, leading to increased cell motility and the formation of invasive structures such as membrane blebs and invadopodia [71–73]. These changes facilitate tumor cell invasion through the extracellular matrix and contribute to metastatic dissemination. Additionally, ROCK signaling regulates epithelial-to-mesenchymal transition (EMT), a key process in tumor metastasis, and is implicated in cancer-associated fibroblast activation and tumor microenvironment remodeling [74]. Elevated ROCK expressions or activity have been observed in various cancers, including breast, prostate, liver, and colorectal cancers, and are often associated with poor prognosis [72,73,75,76]. Pharmacological inhibition of ROCK has been shown to suppress tumor growth, reduce metastasis, and enhance chemosensitivity in preclinical models, indicating its potential as a therapeutic target in oncology [16,77,78].

Currently, several therapeutic strategies combine ROCK inhibitors with chemotherapy or immunotherapy to enhance treatment efficacy [79]. Integrating ROCK inhibitors as adjunctive or neoadjuvant therapies has demonstrated potential in preclinical models. For instance, in pancreatic cancer, fasudil increased gemcitabine uptake and prolonged survival [80]. Y27632 enhanced doxorubicin efficacy in colorectal and melanoma models by promoting antitumor immunity and CD8+ T cell priming [81] and improved bortezomib response in multiple myeloma through modulation of the bone marrow microenvironment [82]. Dual inhibition of ROCK and EGFR emerged as a promising strategy in triple-negative breast cancer, suppressing autophagosome clearance and inducing cancer cell death [83]. In melanoma, ROCK activation is associated with BRAF inhibitor resistance and dedifferentiation [84,85]. In breast cancer, Y27632 decreased PD-L1 expression, promoting T cell activation. Similarly, in uveal melanoma, ripasudil combined with checkpoint inhibitors and photodynamic therapy effectively recruited immune cells [86]. ROCK inhibitors may also function as “migrastatic” preventing metastasis and sensitizing tumors to chemotherapy. Fasudil reduced extravasation in pancreatic cancer and restructured the metastatic niche, improving chemotherapy efficacy [87]. Collectively, these findings underscore the therapeutic potential of ROCK inhibitors as adjuncts to conventional treatments, targeting multiple cancer-related pathways to enhance therapeutic outcomes. Table 1 shows the effects of ROCK inhibitors in several types of cancers.

## 3. ROCK Inhibitors

ROCK inhibitors have been used in clinical settings for nearly three decades. Fasudil, a pan-ROCK inhibitor, was the first ROCK-targeting drug approved for clinical use (1995, Japan), to treat cerebral vasospasm following subarachnoid hemorrhage [88]. Ripasudil (trade name *Glanatec*), a derivative of fasudil identified through a structure-activity screening, exhibits improved potency and selectivity toward ROCK kinases. It was approved in Japan in 2014 for the treatment of glaucoma and ocular hypertension [89]. Furthermore, two additional ROCK-targeting drugs have recently been approved: netarsudil (marketed as *Rhopressa* in the U.S. and *Rhokiinsa* in the EU), which was approved by the FDA in 2017 and by the EMA in 2019 for the treatment of open-angle glaucoma and ocular hypertension [90], and belumosudil (marketed as *Rezurock*), which received FDA approval in 2021 for the treatment of chronic graft-versus-host disease [91].

### 3.1. Fasudil

Fasudil (brand name Eril^®^), also known as ATB877 and HA1077, is a widely used pan-ROCK inhibitor that inhibits both ROCK1 and ROCK2 non-selectively. In humans, it is metabolized mainly into hydroxyfasudil, the functional form with a potency similar to fasudil [103–106]. As a ROCK inhibitor and vasodilator, fasudil is used as a prophylactic treatment to prevent cerebral vasospasm after subarachnoid hemorrhage and has a beneficial efficiency of 74.5% [105]. It was initially developed as a calcium channel antagonist in Japan but subsequently identified as a potent and selective ROCK inhibitor. Approved in 1995, fasudil is administered intravenously to prevent delayed ischemic neurological deficits associated with aneurysmal subarachnoid hemorrhage [107]. The primary mechanism of action involves selective inhibition of ROCK activity, leading to decreased phosphorylation of MLC and enhanced relaxation of vascular smooth muscle cells [108]. Additionally, fasudil enhances endothelial nitric oxide synthase (eNOS) expression, promoting vasodilation via increased nitric oxide production [109]. Beyond its initial vascular indications, emerging evidence suggests fasudil has therapeutic potential in neurodegenerative diseases such as ALS and PD, demonstrating improved motor function and survival in animal models [68,110–112]. Thus, fasudil remains a critical pharmacological tool for ROCK inhibition, with expanding therapeutic indications and formulation strategies holding promises for diverse clinical applications.

### 3.2. Ripasudil

Ripasudil (K-115), marketed under the brand name Glanatec^®^, was primarily developed for ophthalmological applications. Ripasudil was initially discovered and synthesized by Kowa Company, Ltd. (Nagoya, Japan) and approved in Japan in 2014 for treating glaucoma and ocular hypertension [113, 114]. Unlike fasudil, ripasudil is formulated as topical eye drops, enabling localized drug delivery with minimal systemic side effects. Mechanistically, ripasudil selectively binds to and inhibits ROCK, reducing actomyosin contractility in trabecular meshwork cells, thereby enhancing aqueous humor outflow and lowering intraocular pressure (IOP) [89,115]. Clinical studies demonstrated that ripasudil significantly reduces IOP in patients with primary open-angle glaucoma and ocular hypertension, showing efficacy comparable to conventional treatments with a favorable safety profile [116]. Additionally, ripasudil has shown potential in corneal endothelial regeneration, with studies indicating enhanced endothelial wound healing and cell proliferation, highlighting possible broader therapeutic applications in ophthalmology [117]. Thus, ripasudil represents a promising ROCK inhibitor with targeted ophthalmic applications, expanding the potential for corneal tissue repair.

### 3.3. Netarsudil

Netarsudil (AR-13324), marketed as Rhopressa^®^, is a novel ROCK inhibitor developed primarily for treating glaucoma and ocular hypertension. Netarsudil was developed by Aerie Pharmaceuticals (Durham, NC, USA) and approved by the U.S. Food and Drug Administration (FDA) in 2017 [90, 118, 119]. It also functions by binding competitively to the ATP-binding pocket of ROCK, inhibiting kinase activity, promoting relaxation of the trabecular meshwork, and increasing aqueous humor outflow, ultimately reducing IOP. In addition, netarsudil uniquely targets the norepinephrine transporter (NET), which further enhances its IOP-lowering capability through decreased aqueous humor production and lowered episcleral venous pressure [120]. Clinical studies have demonstrated its effectiveness as monotherapy and in combination with other medications, such as latanoprost [121,122], and it is administered as a topical ophthalmic solution. Netarsudil is particularly noted for its ability to lower IOP independent of baseline pressure, making it effective even in patients with low baseline IOP or normal tension glaucoma. Common side effects include conjunctival hyperemia, instillation-site pain, and corneal verticillata, which are typically manageable and mild in severity [123].

### 3.4. Belumosudil (KD025)

Belumosudil is a highly selective oral inhibitor of ROCK2, which has an IC50 of ROCK2 at 105 nM and ROCK1 at 24 μM [124], developed by Kadmon Pharmaceuticals (New York, NY, USA) and approved by the U. S. FDA in 2021 under the trade name Rezurock^®^. Distinct from previous ROCK inhibitors, belumosudil exhibits substantial selectivity toward the ROCK2 isoform, minimizing off-target effects related to ROCK1 inhibition and reducing common side effects such as hypotension [125]. The specificity for ROCK2 confers belumosudil’s distinct pharmacological advantages, particularly in diseases driven by pathological fibrosis and inflammation. It has demonstrated efficacy in chronic graft-versus-host disease (cGVHD) [126,127], where selective ROCK2 inhibition attenuates fibrosis by regulating CTGF and modulates regulatory T-cell function through STAT3/STAT5 signaling pathways, resulting in a significant reduction of inflammation and tissue remodeling [15]. Belumosudil is orally bioavailable and typically administered as a tablet. It is the only cGVHD drug targeting inflammation and fibrosis, a hallmark feature of the disease’s late stage. Ongoing clinical studies explore additional therapeutic indications, including idiopathic pulmonary fibrosis, systemic sclerosis, and other autoimmune diseases characterized by ROCK2-driven fibrosis [12,47,91,128].

### 3.5. Preclinical Studies

Numerous preclinical studies have demonstrated the therapeutic potential of ROCK inhibitors across a broad spectrum of diseases.

Y-27632 (Mitsubishi Tanabe Pharma, Osaka, Japan), one of the earliest and most widely used ROCK inhibitors, has been extensively applied in cell culture systems to enhance survival and proliferation, particularly in corneal endothelial cell transplantation and regenerative models of bullous keratopathy [117,129].

GSK269962A (GSK, Marietta, PA, USA), a selective ROCK1 inhibitor, exhibited anti-leukemic effects in murine models of acute myeloid leukemia by inducing apoptosis and inhibiting the ROCK1/c-Raf/ERK signaling axis, thereby reducing leukemic infiltration and prolonging survival [130].

Zelasudil (Redx Pharma, Cheshire, UK), a next-generation ROCK2-preferential inhibitor, has shown anti-fibrotic activity in preclinical models and is currently under investigation in a phase II clinical trial for idiopathic pulmonary fibrosis.

In the field of ophthalmology, several locally acting ROCK inhibitors—Sovesudil, and AR-12286—have been developed and evaluated in preclinical and early clinical models for glaucoma and ocular hypertension [131,132]. Sovesudil effectively lowered intraocular pressure in normal-tension glaucoma, while AR-12286 showed intraocular pressure-lowering effects in patients with exfoliation syndrome [133].

Topical ROCK inhibitors have also been studied in the postoperative setting, showing potential benefit after cataract surgery damage (Y-27632) and Fuchs dystrophy after Descemet stripping only (Ripasudil) [117,134,135]. Fasudil has progressed from preclinical neuroprotective studies to clinical trials for neurodegenerative disorders such as ALS, where it demonstrated good tolerability and suggested efficacy in slowing disease progression [136]. In the cardiovascular and pulmonary systems, ROCK inhibition has been shown to improve vascular function and tissue perfusion in preclinical studies. For example, fasudil improved hemodynamic responses, increased circulating ATP during hypoxia and handgrip exercise in older adults, and reduced pulmonary vascular resistance in animal and early human studies [137]. Collectively, these preclinical findings support the expanding application of ROCK inhibition in fibrotic, vascular, neurodegenerative, and ophthalmic disease contexts.

### 3.6. Therapeutic Limitations

Despite their therapeutic promise, the clinical application of ROCK inhibitors is limited by several key challenges. Firstly, isoform specificity remains a critical issue. ROCK1 and ROCK2 share high sequence homology, particularly in their kinase domains, making it challenging to design isoform-selective inhibitors. Most current inhibitors, such as fasudil and Y-27632, target both ROCK isoforms with similar potency, leading to unintended effects. ROCK1 and ROCK2 exhibit dominant roles in different organs and have different, sometimes opposite functions. Isoform-specific ROCK1 or ROCK2 inhibitors may retain therapeutic efficacy while minimizing side effects to the greatest extent possible. Recent ROCK inhibitors like belumosudil and zelasudil show ROCK2 specificity, but till now there are no drugs targeting ROCK1 only. Secondly, off-target effects are a significant concern. Many ROCK inhibitors like Y-27632 also affect other AGC family kinases, including PKA and PKC [79]. Additionally, fasudil has been shown to inhibit not only ROCK but also PKA, PKB, PKC, PKG, MLCK, CaMKII, and other kinases, although 10 times lower, but still retains relative potent inhibition activities against these kinases [138]. Thirdly, dose-dependent toxicity remains a significant concern, particularly for non-selective inhibitors. High doses of fasudil and Y-27632 are associated with significant hypotension due to systemic vasodilation mediated by vascular smooth muscle relaxation [139]. In cancer therapy, prolonged ROCK inhibition can lead to drug resistance, potentially through compensatory activation of alternative pro-survival pathways such as the PI3K/AKT axis [79]. Addressing these limitations requires the development of isoform-selective inhibitors and targeted delivery systems to enhance therapeutic efficacy while minimizing off-target effects.

## 4. Perspectives

The therapeutic potential of Rho/ROCK signaling inhibition continues to expand across multiple disease domains, supported by a growing body of mechanistic and translational evidence. In ophthalmology, topical ROCK inhibitors have revolutionized the treatment paradigm for glaucoma and corneal endothelial diseases, with emerging evidence supporting their use in regenerative therapies and postoperative recovery. In cardiovascular and metabolic diseases, isoform-specific targeting of ROCK1 or ROCK2 holds promise for improving endothelial function, attenuating fibrosis, and protecting against diabetic complications such as cardiomyopathy and nephropathy. In the nervous system, ROCK inhibition offers a multipronged approach to modulate neuroinflammation, promote axonal regeneration, and potentially delay neurodegenerative processes. ROCK’s role in tumor invasion and the tumor microenvironment in oncology underscores its relevance as a co-target in metastatic disease. Moreover, the success of belumosudil in chronic graft-versus-host disease highlights the immunomodulatory potential of ROCK2-selective inhibition, paving the way for its application in other immune-mediated and fibrotic conditions. Despite these advances, challenges remain in achieving tissue-specific delivery, minimizing off-target effects, and elucidating the context-dependent roles of ROCK isoforms. Future directions will likely include the development of next-generation inhibitors with enhanced selectivity, optimized pharmacokinetics, formulation innovations, and deeper exploration of ROCK’s crosstalk with other signaling pathways in health and disease. Collectively, the Rho/ROCK pathway is a versatile and promising drug target with broad clinical relevance and substantial room for therapeutic innovation.

## Figures and Tables

**Table 1.** Effects of ROCK inhibitors in cancer.

Cancer Type	ROCK Inhibitor(s)	Experimental Model/Context	Functions
Bladder cancer	Y-27632	T24, 5637 and TSGH bladder carcinoma cell lines	Inhibits proliferation and invasion [92,93]
Breast cancer	Y-27632	Bone metastasis model, Syngeneic breast tumor model; breast cancer cells	Reduces bone metastasis by impairing tumor cell motility, Decreases PD-L1 [94,95]
Hepatocellular carcinoma (HCC)	Y-27632	HCC Li7 cells; Orthotopic liver tumor in SCID mice	Blocks actin reorganization and cell motility; decreases intrahepatic metastatic nodules [96]
Lung cancer (NSCLC)	fasudil	A549 non-small cell lung carcinoma cells	Inhibits cell migration [97]
Lung cancer (SCLC)	fasudil	Small-cell lung cancer xenograft in nude mice	Inhibits tumor growth, metastasis, and induces apoptosis [98,98]
Melanoma	fasudil	B16 melanoma 3D culture and C57BL/6 mouse model	Disrupts vasculogenic mimicry, reduces tumor growth [99]
Neuroblastoma	RKI-1447	Neuroblastoma cell lines and xenograft/zebrafish models	Suppresses cell proliferation and viability, induces cell death/apoptosis [100]
Prostate cancer	Y-27632	PC3 prostate carcinoma cells	Inhibits proliferation, migration, and induces apoptosis [76,101,102]
